# Incorporating Interpersonal Synchronization Features for Automatic Emotion Recognition from Visual and Audio Data during Communication

**DOI:** 10.3390/s21165317

**Published:** 2021-08-06

**Authors:** Jingyu Quan, Yoshihiro Miyake, Takayuki Nozawa

**Affiliations:** 1 Department of Computer Science, Tokyo Institute of Technology, Yokohama 226-8502, Japan; quan.j.aa@m.titech.ac.jp (J.Q.); miyake@c.titech.ac.jp (Y.M.); 2 Research Institute for the Earth Inclusive Sensing, Tokyo Institute of Technology, Tokyo 152-8550, Japan

**Keywords:** affective computing, classification, communication, deep neural networks, emotion recognition, interpersonal features, multimodal

## Abstract

During social interaction, humans recognize others’ emotions via individual features and interpersonal features. However, most previous automatic emotion recognition techniques only used individual features—they have not tested the importance of interpersonal features. In the present study, we asked whether interpersonal features, especially time-lagged synchronization features, are beneficial to the performance of automatic emotion recognition techniques. We explored this question in the main experiment (speaker-dependent emotion recognition) and supplementary experiment (speaker-independent emotion recognition) by building an individual framework and interpersonal framework in visual, audio, and cross-modality, respectively. Our main experiment results showed that the interpersonal framework outperformed the individual framework in every modality. Our supplementary experiment showed—even for unknown communication pairs—that the interpersonal framework led to a better performance. Therefore, we concluded that interpersonal features are useful to boost the performance of automatic emotion recognition tasks. We hope to raise attention to interpersonal features in this study.

## 1. Introduction

During communication, emotion recognition skills help us understand the attitude, feeling, and intention of the partner, and therefore guide our behavior to make the communication successful. However, the ability of emotion recognition is different from person to person, and we sometimes fail to recognize the emotion of the interlocutor. This kind of mistake can lead to mutual misunderstandings, impeded communication, and deterioration in relationships [1]. To avoid such failures and improve communication, one solution here is to use the power of machine learning.

Thanks to the significant development in the field of machine learning, recently we have obtained many software programs that can automatically recognize human emotion [2,3,4,5]. Although the methods of automatic emotion recognition emerge, their performance is still unsatisfactory [6,7]. Therefore, we hope to propose a possible method to achieve a better performance.

**Figure 1:**
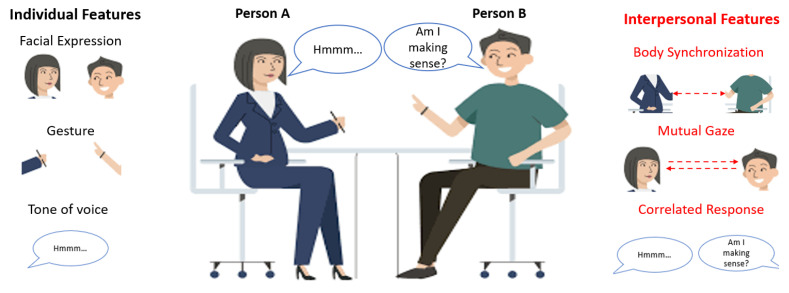
Interpersonal and individual features help humans recognize emotion.

As illustrated in Figure 1, humans recognize others’ emotions through both individual features and interpersonal features. Studies [8,9,10] have shown that the individual features such as facial expression [11,12], gesture [13,14], and tone of the voice [15] help us to recognize others’ emotions. For example, if a man clenches his fist, it may mean he is angry. If a man frowns, it may mean sorrow.

Studies also have shown that interpersonal features such as mutual gaze [16,17], body synchronization [18] and the synchronization of speech [19] will help us to recognize others’ emotions. Here, the interpersonal features used in this study are defined as the interpersonal interaction activities (verbal or nonverbal) that happen consciously or unconsciously during communication. It is important for the emotion recognition task because first- and third-person emotion recognitions will be influenced by these features [20]. For example, during an interaction, if you have a mutual gaze and touch your partner, greater positive emotion will be observed [21]. If the partner synchronizes with your action, the positive emotion will increase [18]. Furthermore, sometimes interpersonal features play a crucial role in recognizing emotion. For example, when one interlocutor is not very expressive, it will be hard to recognize his/her emotion from the individual features only. However, the synchronization of body movement with the interlocutor may help humans recognize the emotion. (E.g., if the synchronization is high, the possibility of positive emotion is high. See [22] for a review.)

However, to the best of our knowledge, most current automatic emotion recognition technologies either only use the individual features or just simply combine individual features to capture interpersonal features (see Related Work below). They overlooked the importance of synchronization features. Therefore, we aim to explore the following questions in this study: Are the interpersonal features, especially time-lagged synchronization features, beneficial for automatic emotion recognition tasks? Here, time-lagged synchronization includes both concurrent (i.e., zero-lag) interpersonal features such as mutual gaze and mirroring of facial expressions, and action–reaction (i.e., lagged) interpersonal features such as utterances and responses or smile to smile.

We addressed this question using the K-EmoCon [23] dataset, a publicly available multimodal dataset of naturalistic conversations with continuous annotation of emotions by the participant themselves, as well as external emotion annotation. Using visual, audio, and audio-visual cross-modal features, respectively, we built two types of emotion recognition models: an individual model and interpersonal model. The individual model serves as a control condition using only individual features. The interpersonal model serves as an experimental condition, using both the individual and interpersonal features. We compared the performance of the models to judge whether interpersonal features are beneficial or not. Based on the findings on the importance of interpersonal features, we hypothesized that the interpersonal models would outperform the individual models with either unimodal or cross-modal features.

## 2. Related Work

Emotion recognition is a challenging task due to the difficulty of discrimination [24] and diverse expression modalities [25]. To solve the challenge of abstraction of emotion, researchers tried to use different features to discriminate different emotions. However, most of them are individual features.

A common feature used in visual modalities is facial expression [26,27,28,29,30,31,32]. Given a raw image, researchers used face detection methods [33,34,35] to find the position of the face first. Then, they cropped the face and extracted the feature of facial expressions. Finally, they fed these features into the classifier [36,37] to obtain the emotion. Some popular methods include DTAGN [38], FN2EN [39], LPQ-SLPM-NN [32], and so on. In addition to facial expression, gestures are also a common feature [40,41,42,43]. The researchers first used pose estimation methods [44,45,46] to obtain the pose of humans. Then, they fed the pose into a classifier to obtain the emotion. Pupil size [47] and gaze [48] are also important features used for recognizing emotion.

For the audio modality, the speech features [49,50,51,52,53] include qualitative features, such as voice quality, harshness, tense and breathy; continuous features, such as energy, pitch, formant, zero-cross rate (ZCR), and speech rate; spectral features, such as Mel-frequency cepstral coefficients (MFCC), linear predictor coefficients (LPC), perceptual linear prediction (PLP), and linear predictive cepstral coefficients (LPCC); Teager energy operator (TEO)-based features, such as TEO-decomposed frequency modulation variation (TEO-FM-Var), normalized TEO autocorrelation envelope area (TEO-Auto-Env), and critical band based TEO autocorrelation envelope (TEO-CB-Auto-Env). Similar to the visual modality, given the raw speech signal, researchers first extracted their desired features such as above, then fed them into the classifier. Different from the above individual features methods, Lin [54], Lee [55], and Yeh [56] tried to use interpersonal features in audio modality to boost the performance of automatic emotion recognition. However, they did not explore whether synchronization will be beneficial or not, which is the main target of this study.

Although researchers have spent decades on emotion recognition tasks using unimodal features, the performance is still not satisfactory. To achieve a better performance, researchers proposed to fuse visual and audio modalities [57,58]. To further improve the performance, others tried to fuse not only the audio and visual modality but also the context modality [59,60]. This fusing strategy improved the performance of the emotion recognition task further, because multimodality can give mutually supplementary information that is missed in the unimodal approaches.

Despite all these efforts, we believe that there still is room for improvement. We were motivated by psychological studies that indicated that humans also use interpersonal features to recognize others’ emotions [16,17,18,19]. According to our best knowledge, although the previous automatic emotion recognition research paid great attention to the individual features, most studies did not pay attention to the interpersonal features, especially the time-lagged synchronization. Therefore, we constructed an interpersonal model in the present study to explore whether interpersonal features are beneficial for emotion recognition or not.

## 3. Methods

**Figure 2:**
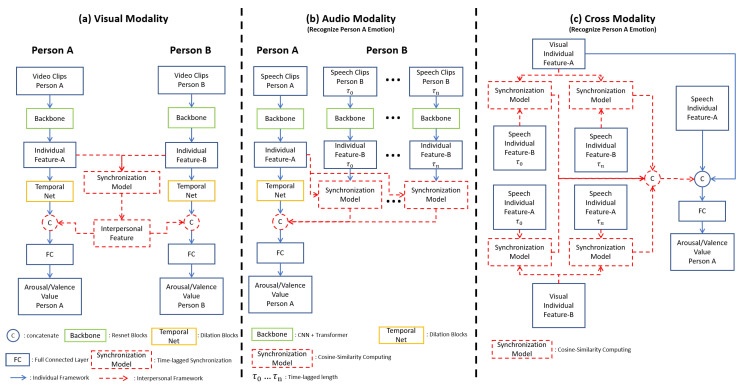
Individual and interpersonal models in visual, audio, and cross-modality.

The present study has two aims. First, we aimed to establish the usefulness of interpersonal features for an emotion recognition task. To achieve this, we constructed two models for comparison. One is the individual model using only individual features. Another one is the interpersonal model using both individual and interpersonal features. The only difference in structure between the two models is that the interpersonal model includes the synchronization model (the red block in Figure 2). Second, we aimed to show the power of interpersonal features in multiple modalities. Therefore, we built the models that use visual, audio, and audio-visual cross-modality features, respectively. Figure 2 shows the general framework of our individual and interpersonal models using visual (Figure 2a), audio (Figure 2b), and cross-modality (Figure 2c). We note that we detected the emotions of both individuals (person A and B) in dyadic communication using visual, audio, and cross-modality. However, to explain our methods concisely, we use the scenario of predicting person A’s emotion as an example.

### 3.1. K-EmonCon Dataset

To compare the individual model with the interpersonal model in different modalities, and to perform our experiments with minimum human intervention, we decided to use the K-EmoCon dataset [23] to test the usefulness of interpersonal features because, to our best knowledge, the K-EmoCon is the *only* dyadic dataset in which the subjects show *spontaneous* emotions during *naturalistic* conversations.

Other datasets are not suitable for our experiments due to posed or induced emotions and limited situation. For example, the IEMOCAP [61] is a popular dataset used for the emotion recognition task. However, IEMOCAP was considered to contain induced emotion and posed emotion by actors. As we aim to achieve the recognition of natural (spontaneous) emotions during dialogue communication, containing induced emotions and especially the posed emotions violates our purpose. Unlike IEMOCAP, the content in K-EmoCon is the natural debate between individuals without professional training in acting, which means it is more like an in-the-wild challenging situation.

**Figure 3:**
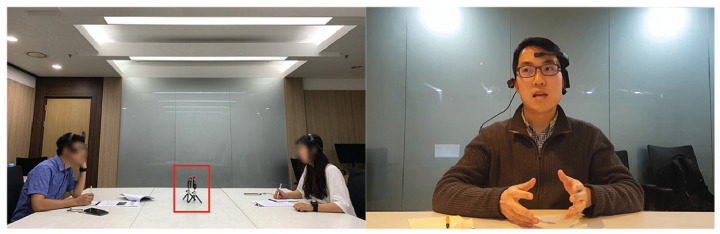
Scenario (**left**) and Sample image (**right**) in K-EmoCon dataset [23].

Figure 3 shows scenario and a sample image in the K-EmoCon dataset. The original K-EmoCon dataset includes 32 participants. However, for the complete audiovisual recording, there are 16 participants (Person IDs: 3, 4, 7, 8, 9, 10, 19, 20, 21, 22, 23, 24, 25, 26, 29, 30) available in the dataset. The 16 participants are paired into eight sessions. For example, Person IDs 3 and 4 are in one session. Each session contains an approximately 10-min-long paired debate on a social issue. The video (frame rate ($N_{V}$): 30 fps) is re-sized into 112 × 112 and records participants’ facial expressions, upper body, and speeches (sampling rate: 22 kHz).

The original K-EmoCon dataset contains emotion annotations by the subjects themselves, by the partner, and by external raters. Since our purpose in this study was to test the utility of interpersonal feature in recognizing subjectively experienced emotions rather than the observed/inferred emotions by others, we decided to use self-reported annotations as the label. Although the K-EmoCon dataset also contains the labels of “cheerful”, “happy”, “angry”, “nervous”, and “sad”, their values are heavily imbalanced (see Figure 3 in [23]) compared to the more normally distributed arousal and valence. Furthermore, arousal and valence are the two affective dimensions of the well-known circumplex model of emotion by James Russell [62], which can cover more subtle changes in emotions. Thus, we used the arousal and valence labels for our emotion recognition task.

Specifically, we chose to use the self-reported arousal and valence which were rated on a five-level scale (from 1: very low to 5: very high) for every 5 s as emotion labels. Therefore, for the recognition of each 5 s segment of emotional state, the original input size of individual video clips ($\left[T_{V}\times N_{V},C_{V},W_{V},H_{V}\right]$) was [5×30,3,112,112], and the original size of individual speech data ($\left[T_{A},C_{A},F_{A}\right]$) was [5,2,22050]. In extracting MFCC features for audio data, we framed the audio data into the same temporal size as the visual data. That is, the temporal dimension for audio data after MFCC was 150, which is equal to $T_{V}\times N_{V}$ (visual temporal dimension). We formalized the emotion recognition as a classification task, similarly to [63], because the annotated emotion labels in K-EmoCon are limited to five-level scale instead of continuous values in an interval. Moreover, the labels changed by steps at intervals of every 5 s instead of changing continuously frame by frame, which made the task more like qualitative task rather than quantitative task.

### 3.2. Visual Modality

#### 3.2.1. Individual Model

Let us begin with the individual model for the visual modality. In general, our individual model includes three stages (Figure 2a).

- The first stage is to feed the individual video clips ($I_{Video}^{A}$ or $I_{Video}^{B}$) into the backbone to extract spatial information and obtain individual features;
- The second stage is to feed the individual features into the Temporal Net to extract temporal information;
- The final stage is to feed the output from the Temporal Net into a fully connected layer to predict the value of arousal or valence. Now, we explain the detail of each component.

**Figure 4:**
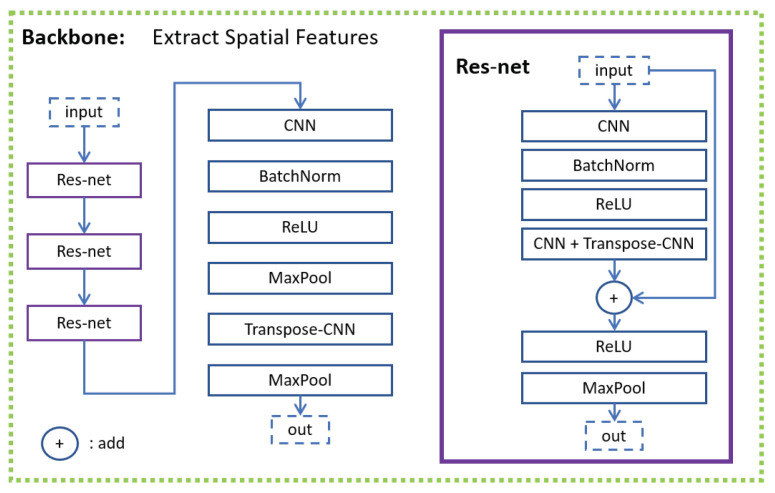
Backbone for visual modality.

**Figure 5:**
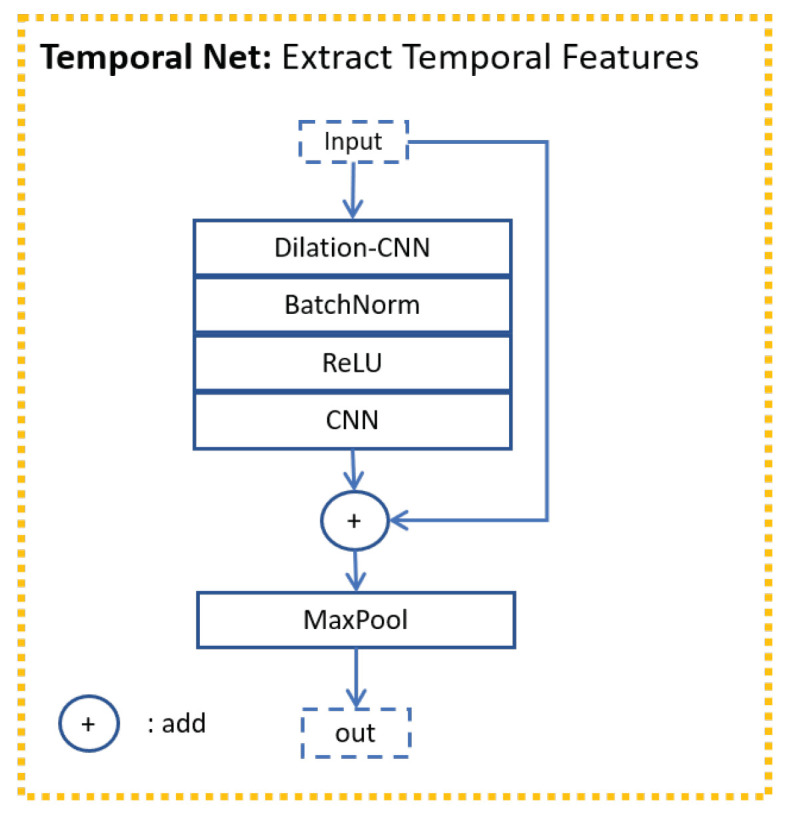
Temporal Net for visual modality.

The backbone (Figure 4) for visual modality includes a convolutional neural network (CNN) [64] and transpose CNN [65]. It is a structure similar to Resnet [66]. A CNN was used to extract the local information first. A transpose CNN was used to extract further information and reshape the output to make its size equal to the size of the input. To obtain the general information, max-pooling was used to down-sample and summarize the local information. In the backbone, CNN plus transpose CNN were used for a total of four times. The first three times were used in the Resnet structure (purple line in Figure 4) to deepen our network because the mapping from the input features to emotional states requires heavy nonlinear transformation. The fourth time is slightly different from the first three times. The CNN was not connected with the transpose CNN directly. The max-pooling was inserted between the CNN and transpose CNN to reduce computing complexity. The Temporal Net (Figure 5) here is a structure similar to the temporal CNN [67]. Dilation CNN and Resnet were used to extract temporal information. As the backbone for visual modality is deep enough, only one layer was used in Temporal Net to prevent overfitting.

#### 3.2.2. Interpersonal Model

**Figure 6:**
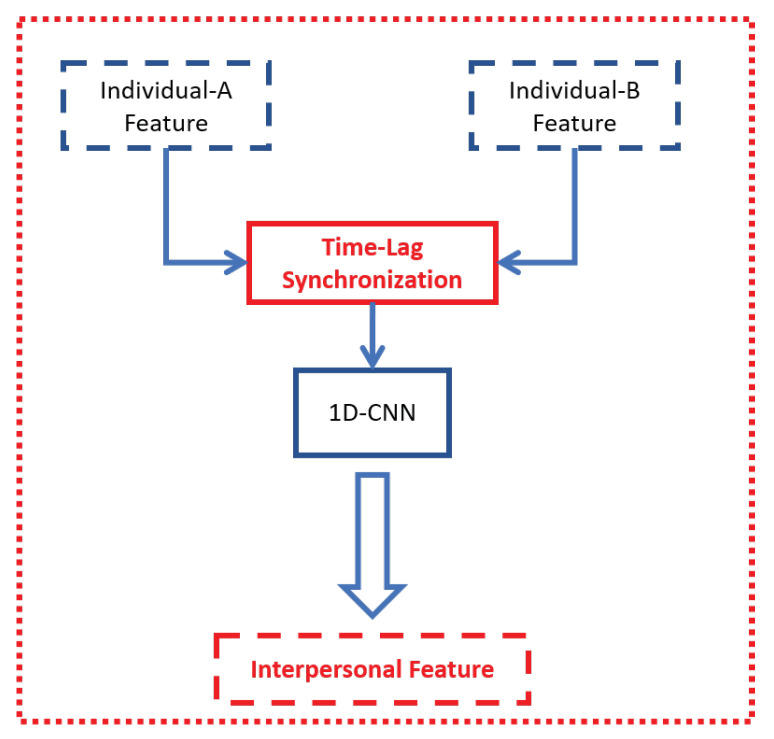
Synchronization model for visual modality.

Next, we used the task of predicting Person A’s emotion as an example to explain the process to obtain interpersonal feature $Y_{Video}^{I}$. (When predicting person B’s emotion, the process is symmetrical.) In general, the interpersonal feature $Y_{Video}^{I}$ was obtained by feeding the respective individual features ($X_{Video}^{A}$ and $X_{Video}^{B}$) into the synchronization model $M_{S}$ as shown in Figure 6. Specifically $X_{Video}^{A}$ as shown in Equation (1). As for person B, we obtained $X_{Video}^{B}$ with a different backbone model $M_{Video}^{B}$ as shown in Equation (2). Then, the pair of individual features ($X_{Video}^{A}$ and $X_{Video}^{B}$) were fed into the synchronization model $M_{S}$ (Equation (3)) to obtain interpersonal features $Y_{Video}^{I}$. Finally, the interpersonal features were combined with individual features and fed into the fully connected layer to predict the value of emotion.

(1) $$X_{Video}^{A}=M_{Video}^{A}\left(I_{Video}^{A}\right)$$

(2) $$X_{Video}^{B}=M_{Video}^{B}\left(I_{Video}^{B}\right)$$

(3) $$Y_{Video}^{I}=M_{S}\left(X_{Video}^{A},X_{Video}^{B}\right)$$

We note that $M_{Video}$ processed the spatial dimension, which means it conserves the temporal order of video clips. For example, the size of the original input $I_{Video}$ is [T,C,W,H], where *T* represents the time length of clips, *C* represents the RGB channel, *W* represents the frame image width, and *H* represents the frame image height. After the processing of $M_{Video}$, the size of $X_{Video}$ is [T,F], where *T* keeps the same and *F* represents the length of the individual feature vector. The specific values used in the experiment are specified in Section 3.1.

The synchronization model consists of two parts. The first part is the computation of time-lagged synchronization similarly to the time-lagged detrended cross-correlation analysis (DCCA) cross-correlation coefficient computing process [68]. The second part is to use 1D CNN to further extract information.

The detailed algorithm is shown in Algorithm 1. When computing time-lagged synchronization $Y_{Video}^{S}$, the individual features ($X_{Video}^{A}$ and $X_{Video}^{B}$) were first divided into several temporal blocks (*R* and $R^{\prime }$). The number of blocks is $n_{block}$. Then the cosine similarity was computed with *R* and $R^{\prime }$ as shown in Equation (4). During this computing, the decay weight β was used for the output to emphasize the time-lagged feature. We design $\alpha =1-\frac{i_{\tau }}{n_{block}}\beta$, because as $i_{\tau }$ increases, the α decreases, which is similar to our memory that will forget information along with time. Finally, the mean of the output ($Out_{block}$) was calculated. The calculated **Means** ($Out_{block}$) were combined as $Y_{Video}^{S}$. Specifically, the size of $Y_{Video}^{S}$ is $\left[N_{o},L\right]$, where $N_{o}$ represents the number of calculated $Out_{block}$ and *L* represents the features length for each $Out_{block}$.

**Algorithm 1** Time-Lagged Synchronization**Input:** Individural Features $X_{Video}^{A}$, $X_{Video}^{B}$**Parameters:** Time block size *n*, Time lag length τ, Decay weight β, Clips time length *N*     $Y_{Video}^{S}=\left\{\right\}$     **for**
*i*
**init** 0 **to**
N−τ−n
**by**
*n*
**do**        $Out_{block}=\left\{\right\}$        $R=X_{Video}^{A}\left(i:i+n\right)$        $n_{block}=\frac{N-(i+n)}{\tau }$        **for**
$i_{\tau }$
**init** 0 **to**
$n_{block}$
**by** 1 **do**            $R^{\prime }=X_{Video}^{B}\left(i+\tau \times i_{\tau }:i+\tau \times i_{\tau }+n\right)$            $\alpha =1-\frac{i_{\tau }}{n_{block}}\beta$            $Out_{block}=Out_{block}\cup \mathrm{CosineSimilarity}\left(R,R^{\prime }\right)\times \alpha$        **end for**        $Y_{Video}^{S}=Y_{Video}^{S}\cup \mathrm{Mean}\left(Out_{block}\right)$
**end for**

**return**
$Y_{Video}^{S}$


(4) $$\mathrm{CosineSimilarity}\left(R,R^{\prime }\right)=\frac{R\cdot R^{\prime }}{∥R∥∥R^{\prime }∥}$$

To further extract the information between each $Out_{block}$, the 1D CNN was used to obtain interpersonal features $Y_{Video}^{I}$.

### 3.3. Audio Modality

#### 3.3.1. Preprocess

The mixture of speech from two speakers in a single audio data file brings a challenge to the audio modality. To overcome this challenge, several preprocessing steps were performed to obtain individual speech features. Given the obtained speech features, we shifted them by different time lengths, because we aimed to capture the action–reaction relationship between the speaker and interlocutor. We predicted Person A’s emotion here as an example. (When predicting person B’s emotion, the process is symmetrical.) Specifically, first, we manually segmented the raw audio data ($I_{Audio}$) to obtain each speaker’s data ($I_{Audio}^{A}$ and $I_{Audio}^{B}$) as shown in Equation (5).

(5) $$\begin{array}{c} I_{Audio}=I_{Audio}^{A}\cup I_{Audio}^{B}\\ ⌀=I_{Audio}^{A}\cap I_{Audio}^{B} \end{array}$$

Then, to synthesize the interpersonal features between $I_{Audio}^{A}$ and $I_{Audio}^{B}$, we shifted person B’s individual speech data $I_{Audio}^{B}$ with different time lengths $\tau_{i}$. For example, $I_{Audio}^{A}$ represents the audio data from time 0 to 5 s, and the $I_{Audio}^{B}$ is from time 0 s to 5 s. If we shift time-lagged length $\tau_{i}$, the shifted $I_{Audio}^{B_{\tau_{i}}}$ will be the data from time $0-\tau_{i}$ to $5-\tau_{i}$. Finally, we extracted MFCC features for $I_{Audio}^{A}$ and $I_{Audio}^{B_{\tau_{i}}}$ to obtain individual speech features ($F_{Audio}^{A}$ and $F_{Audio}^{B_{\tau_{i}}}$) in Figure 2b.

#### 3.3.2. Individual Model

**Figure 7:**
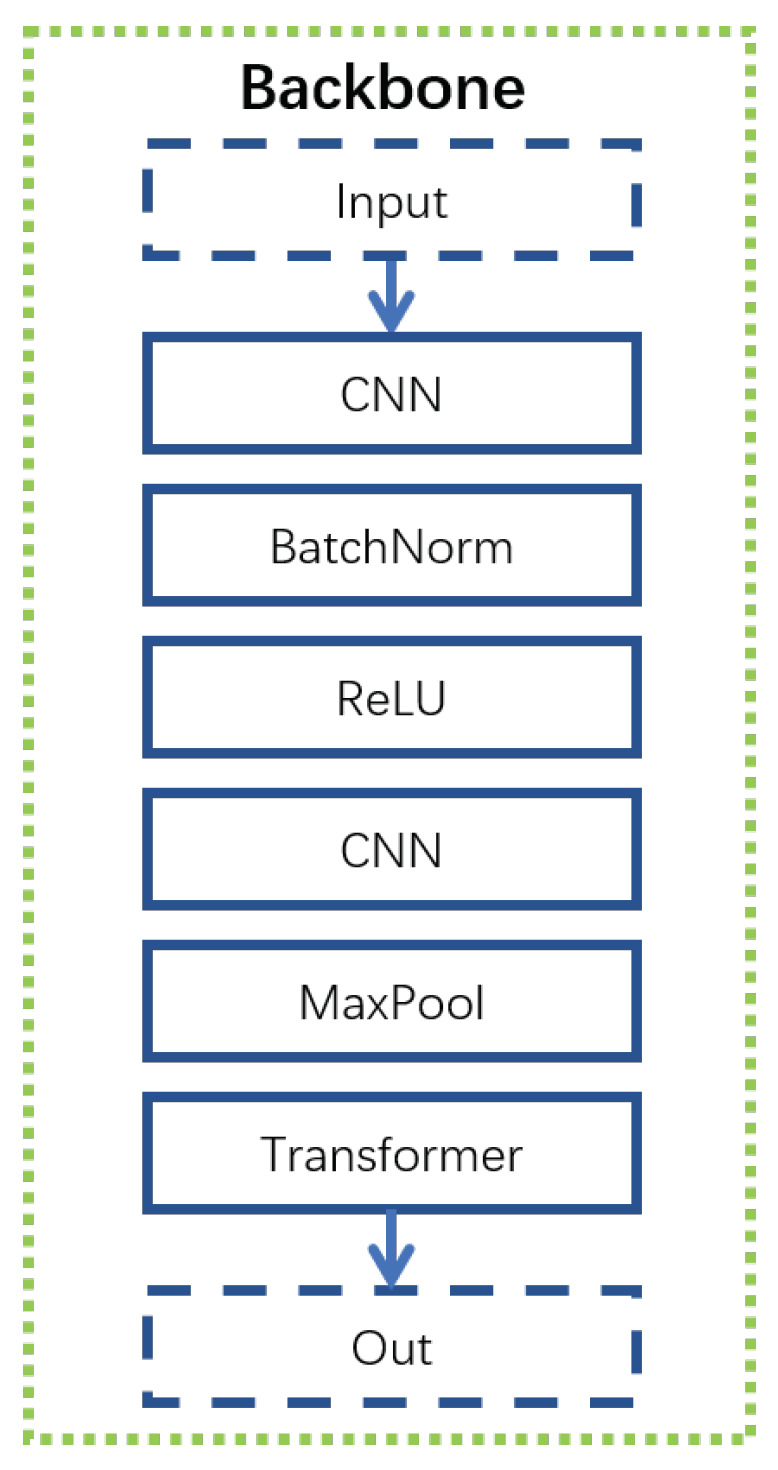
Backbone for audio modality.

Similar to the visual modality, we built both the individual model and interpersonal model (Figure 2b). The individual model is similar to the visual modality. The rough information is extracted by the backbone. Then, the temporal information is further extracted by the Temporal Net (Figure 5). The backbone here is CNN plus Transformer [69] as shown in Figure 7. Specifically, we use da kernel size of (3,1) for the first layer, and the kernel size of (1,1) for the second layer. The different kernel size helps our model extract more different degree local information. With a two-layer CNN, the max-pooling was used to summarize the local information. The output was fed into the Transformer to extract further information. The Temporal Net is the same as the visual modality.

#### 3.3.3. Interpersonal Model

For the interpersonal model, first, the backbone $M_{Audio}^{A}$ was used to obtain $X_{Audio}^{A}$ individual features of the target speaker (Person A in Figure 2b). Then, the backbone $M_{Audio}^{B_{\tau_{i}}}$ was used to obtain $X_{Audio}^{B_{\tau_{i}}}$. $X_{Audio}^{B_{\tau_{i}}}$ are different time-lagged individual speech features of the interlocutor (Person B in Figure 2b). Next, the cosine similarity between the speaker feature vector $X_{Audio}^{A}$ and different time-lagged interlocutor vectors $X_{Audio}^{B_{\tau_{i}}}$ was computed to obtain similarity vectors $Y_{Audio}^{S_{\tau_{i}}}$ as shown in Equation (6).

(6) $$Y_{Audio}^{S_{\tau_{i}}}=\mathrm{CosineSimilarity}\left(X_{Audio}^{A},X_{Audio}^{B_{\tau_{i}}}\right)$$

These similarity vectors were combined as $Y_{Audio}^{S}$ with a decay weight α as shown in Equation (8). The $Y_{Audio}^{S}$ was fed into the CNN to obtain our target—interpersonal features $Y_{Audio}^{I}$. The decay weight α here was calculated with decay parameter β as Equation (7). As $\tau_{i}$ increases the $\alpha_{i}$ will decrease. We also used 1−β to represent the a priori knowledge, which makes sure the 1−β percent of $Y_{Audio}^{S_{\tau_{i}}}$ will contribute.

(7) $$\alpha_{i}=1-\beta +\beta e^{-\tau i}$$

(8) $$Y_{Audio}^{S}=\alpha_{0}Y_{Audio}^{S_{\tau_{0}}}\cup \alpha_{1}Y_{Audio}^{S_{\tau_{1}}}\cup \ldots \cup \alpha_{n}Y_{Audio}^{S_{\tau_{n}}}$$

Finally, the interpersonal features were combined with extracted individual features and fed into a fully connected layer to obtain the emotion value of the target speaker.

### 3.4. Cross Modality

#### 3.4.1. Individual Model

The cross-modality is similar to visual and audio modality, including individual models and interpersonal models (Figure 2c). For the individual model, individual features were extracted from the structure of visual and audio modality as in Section 3.2 and Section 3.3. Then the two modality features were combined and fed into a fully connected layer to predict emotion value.

#### 3.4.2. Interpersonal Model

For the interpersonal model, we incorporated both the audio-visual interpersonal feature $Y_{AV}$ and visual-audio interpersonal feature $Y_{VA}$. We used the prediction of Person A’s emotion as an example.

The audio-visual interpersonal features $Y_{AV}^{S_{\tau_{i}}}$ were obtained by computing cosine-similarity between interlocutor visual modality $X_{Video}^{B}$ and time-lagged speaker audio features $X_{Audio}^{A_{\tau_{i}}}$ as shown in Equation (9). Then, $Y_{AV}^{S_{\tau_{i}}}$ were combined together with decay weight α to obtain visual-audio interpersonal feature $Y_{AV}^{S}$ as shown in Equation (10).

(9) $$Y_{AV}^{S_{\tau_{i}}}=\mathrm{CosineSimilarity}\left(X_{Video}^{B},X_{Audio}^{A_{\tau_{i}}}\right)$$

(10) $$Y_{AV}^{S}=\alpha_{0}Y_{AV}^{S_{\tau_{0}}}\cup \alpha_{1}Y_{AV}^{S_{\tau_{1}}}\cup \ldots \cup \alpha_{n}Y_{AV}^{S_{\tau_{n}}}$$

The visual-audio interpersonal features $Y_{VA}^{S_{\tau_{i}}}$ were obtained by computing the cosine-similarity between speaker visual modality $X_{Video}^{A}$ and time-lagged interlocutor audio features $X_{Audio}^{B_{\tau_{i}}}$ as shown in Equation (11). Then, $Y_{VA}^{S_{\tau_{i}}}$ were combined together with decay weight α to obtain visual-audio interpersonal feature $Y_{VA}^{S}$ as shown in Equation (12).

(11) $$Y_{VA}^{S_{\tau_{i}}}=\mathrm{CosineSimilarity}\left(X_{Video}^{A},X_{Audio}^{B_{\tau_{i}}}\right)$$

(12) $$Y_{VA}^{S}=\alpha_{0}Y_{VA}^{S_{\tau_{0}}}\cup \alpha_{1}Y_{VA}^{S_{\tau_{1}}}\cup \ldots \cup \alpha_{n}Y_{VA}^{S_{\tau_{n}}}$$

Finally, these two interpersonal features ($Y_{AV}^{S}$ and $Y_{VA}^{S}$) were combined with individual features ($X_{Video}^{A}$ and $X_{Audio}^{A}$) and fed into the final layer to predict emotion value.

We note that the computing of the cosine-similarity requires the data to share the same shape in both temporal and feature dimensions. For example, the size of a visual individual feature of speaker $X_{Video}^{A}$ is $\left[T_{V},F_{V}\right]$, and the size of an audio individual feature of interlocutor $X_{Audio}^{B}$ is $\left[T_{A},F_{A}\right]$. The temporal dimension of the individual feature is the same ($T_{V}=T_{A}$) via the neural network that we built. The feature dimension of the individual feature of different modalities was reshaped to the same size *F* with interpolating methods such as Equation (13). Thus, the size of the feature ($X_{Video}^{A}$ and $X_{Audio}^{B}$) satisfied the required conditions $T_{V}=T_{A}$ and $F_{V}^{interpolate}=F_{A}^{interpolate}$.

(13) $$F=\mathrm{interpolate}\left(F_{A}\right)=\mathrm{interpolate}\left(F_{V}\right)$$

## 4. Experiment

### 4.1. Methods Implementation

Table 1 shows the recommended hyperparameters used in the experiment. Specifically, we used stochastic gradient descent (SGD) with momentum as an optimizer. As for the learning schedule, we used cosine annealing (maximum learning rate is 0.01, the minimum learning rate is 0.00001). The β is the decay parameter explained in Section 3. To avoid overfitting, we also used a trick called the flood level [70], which was expressed by *b*. All the models were trained from scratch.

Both the main experiment and supplementary experiment were conducted on a laptop with Intel Core i7-9750H CPU 2.60GHz, 16GB RAM, NVIDIA GeForce RTX 2070 with Max-Q Design and with an operating system Ubuntu.

### 4.2. Main Experiment

#### 4.2.1. Setup

In the main experiment, we split the dataset (985 segments) along with the emotion value into train set and test set randomly. The percentage of train-dataset is 70% and the number of emotion values is five. The main experiment is formulated as a speaker-dependent task, which is similar to [71,72].

It is common that, during usual communication, the emotion value stays in a moderate range most of the time and rarely gets into extreme states, which leads to the imbalanced data problem in our experiment. To solve the imbalanced distribution, several methods could be used such as data re-sampling approaches [73,74,75], class-balanced losses [76,77,78], and so on. Some of the literature has proved that resampling methods can improve the accuracy of class-imbalanced datasets [79]. Therefore, we chose to use data re-sampling approaches to obtain a total of 1000 segments (200 segments × 5 scale value) as training data, of which size is similar to the original dataset. For example, the number of the first class in the total dataset is $D_{C1}^{Total}=100$. Then, the number of the first class in the training dataset is $D_{C1}^{Train}=70$, and the number of the first class in the test dataset is $D_{C1}^{Test}=30$. Finally, after a random re-sampling, the number of the first class in the training dataset is 200.

To compare the interpersonal model with the individual model, we used accuracy as an evaluation metric like most studies. However, due to the imbalance of the test dataset, sometimes the accuracy cannot serve as a great evaluation method to compare the performance. Therefore, we also used the macro-f1-score and unweighted average recall (UAR) as additional evaluation metrics.

#### 4.2.2. Baseline

Although our main purpose is to show the benefit of including interpersonal features, we hope to evaluate our proposed models comprehensively. However, as K-EmoCon is a new dataset, we cannot find suitable methods to compare with our methods directly. Therefore, we re-implemented a popular method called Hierarchical Fusion (HFusion) proposed by Majumder et al. [59]. We compared our individual models with HFusion in visual, audio, and cross (visual-audio) modality, respectively. To make the comparison fair, the setup used for HFusion was the same as the setup used for our models.

Table 2 shows the comparison of results between our individual models and HFusion. Except for the audio valence accuracy, all the results of the individual model were better than HFusion. Moreover, the better f1-score and recall of individual model showed that the better audio valence accuracy of HFusion is because it classified most samples as majority class, which means the individual model was generally better even for predicting valence using audio modality. Therefore, we concluded that our proposed models are effective in the K-EmoCon dataset.

#### 4.2.3. Result

Table 3 shows the test results of performance for the individual and interpersonal models using visual modality to predict arousal and valence. The performance of the interpersonal model was better than the individual model in all target variables and performance metrics. More specifically, the superiority of the interpersonal model was not restricted by the dimension of the emotion, because its performance was better than the individual model both for arousal and valence dimension. The superiority of the interpersonal model was also not restricted by the evaluation metrics because its performance was better than individual model both in terms of accuracy, f1-score, and recall. Therefore, we concluded that interpersonal features are beneficial for emotion recognition in the visual modality.

Table 4 shows the performances of the individual and interpersonal models using audio modality features. As the audio modality included many silent segments, which did not provide useful information for the recognition task, the entire performance of the model using audio modality was lower compared with the visual modality. However, the results here showed that all of the performances of the interpersonal model was higher than the individual model regardless of evaluation metrics and emotion dimension. Therefore, we concluded that the interpersonal features are beneficial for emotion recognition in the audio modality.

Table 5 shows the performance results for the individual and interpersonal models using audio-visual cross-modality. The results again showed that the interpersonal model exhibited a better performance. However, some results of cross-modality were lower than the visual or audio modality, which may violate our intuition. We thought it could be due to two reasons. One is overfitting because we found that the training accuracy and f1-score of cross-modality is higher than other modality. Another is the flaw of audio modality data because the audio data included too much silence.

#### 4.2.4. Discussion

**Figure 8:**
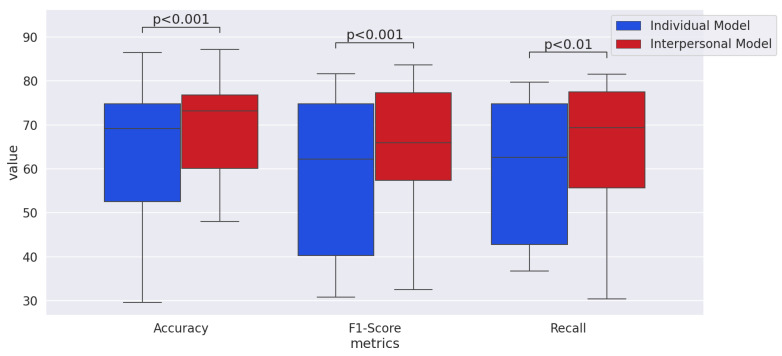
Comparison of the performance metrics between the individual and interpersonal models in the main experiment.

To statistically test whether the interpersonal model significantly outperformed the individual model, we used a two-tailed Wilcoxon signed-rank test, which was also used in [80]. As shown in Figure 8, we pooled the accuracy and f1-score values for all the modalities and emotional dimensions to compare them between the interpersonal model and individual model. The *p*-value for accuracy and f1-score was less than 0.001. The *p*-value of comparing recall between the interpersonal model and individual model was less than 0.01. Thus, we concluded the outperformance of the interpersonal model is significant.

Taken together, we found that interpersonal features are beneficial for automatic emotion recognition regardless of different modalities, different emotion dimensions, and different evaluation metrics. However, in the main experiment, the same individuals contributed to both the training and test data (speaker-dependent task), which means we do not know whether interpersonal features are beneficial for new, unknown samples (speaker-independent task). Therefore, to test the generalization of our hypothesis that interpersonal features are beneficial for even unknown communication groups, we cover the supplementary experiment in the following section.

### 4.3. Supplementary Experiment

#### 4.3.1. Setup

In the supplementary experiment, the percentage of the training dataset was around 75%. Specifically, the training dataset consisted of data from twelve people. The test dataset consisted of the remaining four people. Specifically, the 12 participant IDs for training data were 3, 4, 7, 8, 9, 10, 19, 20, 21, 22, 23, and 24. The remaining four participants, IDs 25, 26, 29, and 30, were used for the test data. We note that there was no particular rule in assigning IDs to participants in the K-EmoCon dataset. Therefore, there is no obvious cause to introduce selection bias.

We faced a problem of imbalanced data in the supplementary experiment because the distribution of emotion labels in the training data and testing data was imbalanced. As an extreme situation, some emotion values in the testing data were not included in the training data, which we did not face in the main experiment. For example, the arousal value 1 was in the training data, while there was no arousal value equal to 1 in test data. To solve this, we collapsed the emotion values into two levels (values from 1 to 3 were put into the low level; values from 4 to 5 were put into the high level). However, even after collapsing into two levels, the problem of imbalanced data still exists. Therefore, we also used the re-sampling methods to obtain 800 samples as training data (400 segments × 2 levels). In addition, we decided to use the F1-score and UAR as evaluation metrics here, because accuracy cannot reflect the true performance of the classifier for such imbalanced data.

#### 4.3.2. Result

Table 6 shows all the f1-score results of the interpersonal model is better than the individual model regardless of modality. Table 7 shows the recall results of the interpersonal model are better than the individual model except for the Visual-Valence and Cross-Arousal results. We will discuss these result in detail according to each modality below.

For the visual modality, although the interpersonal model outperformed individual model in terms of f1-score (Table 6), it showed a worse recall result for the valence dimension (Table 7). We inspected the distributions of the dataset to explore the cause of the difference and found the distribution of valence labels was less balanced in training data and test data.

For the audio modality, as Table 6 shows, all of the results of the interpersonal model are better than the individual model. However, this time, the difference between the valence dimension and arousal dimension was negligible. This can be explained by the fact that the audio data included too much silent part, which would have suppressed the performance of the interpersonal model. Too many silent parts may also have affected the recall result in Table 7. Specifically, the variance of the recall for arousal of the interpersonal model result was very high.

For the cross-modality, Table 6 showed the power of interpersonal features again. We found that the performance boost for the valence dimension was larger than that for the arousal dimension. We also found, similarly to the main experiment, that the result of cross-modality was sometimes lower than visual or audio modality. This could be due to overfitting and the flaw in audio modality data. These two possible problems may also explain the slightly lower recall result of the interpersonal model in the arousal dimension in comparison to the individual model in Table 7.

#### 4.3.3. Discussion

**Figure 9:**
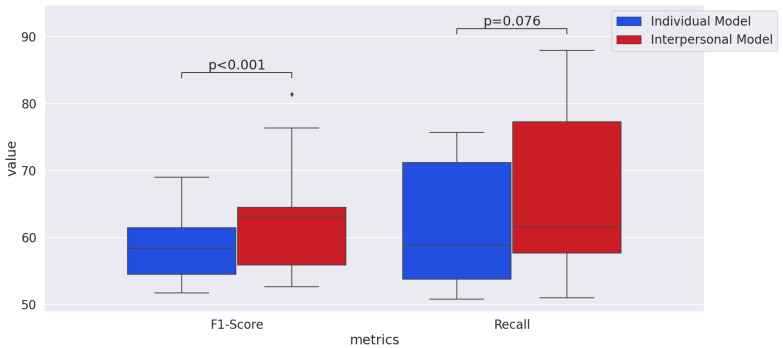
Comparison of the performance metrics between the individual and interpersonal models in the supplementary experiment.

The lower recall result of the interpersonal model in Visual-Valence, and Cross-Arousal may bring up the question regarding whether the interpersonal model outperformed the individual model. We tested this question with a two-tailed Wilcoxon signed-rank test as shown in Figure 9. When we pooled the recall values for all the modalities and emotional dimensions and compared them between the interpersonal model and individual model, the *p*-value was 0.076. Although it is slightly greater than 0.05, it is less than 0.1. Further, comparing the f1-scores between the interpersonal model and the individual model, the *p*-value was less than 0.001, which means that, according to the f1-score, the interpersonal model significantly outperformed the individual model. Therefore, we concluded the interpersonal model was overall better than the individual model, which means interpersonal features are beneficial for automatic emotion recognition even with unknown communication pairs.

## 5. Conclusions

Inspired by the fact the humans recognize emotion via individual features and interpersonal features, we explored whether interpersonal features are beneficial for automatic emotion recognition in this study. Specifically, we constructed the individual model and interpersonal model in visual, audio, cross-modality respectively. Then, we compared these two models using the K-EmoCon dataset with the main experiment and supplementary experiment. Our main experiment results showed that the performance of the interpersonal model was higher than the individual model. Our supplementary experiment results showed—even for unknown communication pairs—that the interpersonal model outperformed the individual model. Therefore, we advocate incorporating interpersonal features for automatic emotion recognition in communication settings.

The framework used in this study was a “black box”. We cannot identify what specific synchronization contributed to better emotion recognition performance. The “black box” nature impeded us from further improving the algorithm, and more importantly impeded us from understanding the mechanism about how humans recognize emotion in nature. In the future, we hope to resolve this issue with the eXplainable Artificial Intelligence (XAI) approach [81].

## Figures and Tables

**Table 1:** Recommended hyperparameters.

	Learning-Rate	Momentum	Epochs	Batch-Size	*b*	β
Value	0.01–0.00001	0.07	20	16	0.01	0.01

**Table 2:** Comparison in terms of accuracy, f1-score, and recall of HFusion [59] with our individual models for visual, audio, and cross-modality.

Modality		Accuracy		F1-Score		Recall	
		Arousal	Valence	Arousal	Valence	Arousal	Valence
Visual	HFusion	62.58±1.51	66.57±9.86	58.09±3.85	60.05±8.39	54.35±6.41	57.02±9.08
	Individual Model	**72.15** ± 2.01	**73.63** ± 9.54	**72.68** ± 1.57	**68.76** ± 10.28	**70.10** ± 6.04	**64.69** ± 12.98
Audio	HFusion	36.91±2.68	**49.74** ± 4.29	26.77±1.04	27.73±2.19	27.32±0.69	27.24±4.69
	Individual Model	**41.60** ± 7.04	42.04±12.51	**36.02** ± 1.57	**36.22** ± 5.43	**40.51** ± 0.64	**39.99** ± 3.29
Cross	HFusion	69.30±2.18	71.28±8.86	69.25±0.12	65.39±12.46	65.76±2.42	61.76±12.49
	Individual Model	**72.48** ± 4.36	**79.17** ± 7.36	**71.19** ± 5.18	**70.03** ± 11.68	**69.33** ± 5.05	**70.50** ± 9.25

**Table 3:** The main experiment result for visual modality.

Model	Accuracy		F1-Score		Recall	
	Arousal	Valence	Arousal	Valence	Arousal	Valence
Individual	72.15±2.01	73.63±9.54	72.68±1.57	68.76±10.28	70.10±6.04	64.69±12.98
Interpersonal	**73.83** ± 2.68	**79.34** ± 6.86	**74.21** ± 2.53	**71.68** ± 11.13	**72.46** ± 5.20	**69.28** ± 12.25

**Table 4:** The main experiment result for audio modality.

Model	Accuracy		F1-Score		Recall	
	Arousal	Valence	Arousal	Valence	Arousal	Valence
Individual	41.60±7.04	42.04±12.51	36.02±1.57	36.22±5.43	40.51±0.64	39.99±3.29
Interpersonal	**53.02** ± 2.68	**54.80** ± 6.81	**49.61** ± 2.16	**47.08** ± 14.60	**47.29** ± 4.36	**45.09** ± 14.71

**Table 5:** The main experiment result for cross-modality.

Model	Accuracy		F1-Score		Recall	
	Arousal	Valence	Arousal	Valence	Arousal	Valence
Individual	72.48±4.36	79.17±7.36	71.19±5.18	70.03±11.68	69.33±5.05	70.50±9.25
Interpersonal	**76.68** ± 1.17	**80.52** ± 6.69	**74.73** ± 4.58	**71.45** ± 12.24	**74.46** ± 3.02	**79.07** ± 1.68

**Table 6:** The Supplementary Experiment Result (F1-Score).

Model	Visual		Audio		Cross	
	Arousal	Valence	Arousal	Valence	Arousal	Valence
Individual	55.76±3.09	65.41±3.61	56.62±2.04	57.72±3.57	53.63±1.96	61.33±5.49
Interpersonal	**59.19** ± 5.88	**71.70** ± 9.65	**59.76** ± 4.33	**59.94** ± 3.89	**55.30** ± 2.68	**70.37** ± 5.95

**Table 7:** The Supplementary Experiment Result (Recall).

Model	Visual		Audio		Cross	
	Arousal	Valence	Arousal	Valence	Arousal	Valence
Individual	53.53±0.42	**73.41** ± 2.26	56.32±4.61	63.18±0.32	**56.30** ± 5.53	65.12±8.34
Interpersonal	**57.40** ± 0.52	70.6 ± 10.29	**69.46** ± 17.44	**64.23** ± 1.37	55.54 ± 4.54	**82.01** ± 5.91

## Data Availability

Link to K-EmoCon dataset: https://zenodo.org/record/3814370#.YNFpjkzTZ7M (accessed on 22 June 2021).

## References

[1] Planalp S. (1999). Communicating Emotion: Social, Moral, and Cultural Processes.

[2] Hazarika D., Poria S., Zadeh A., Cambria E., Morency L.P., Zimmermann R. Conversational memory network for emotion recognition in dyadic dialogue videos. Proceedings of the 2018 Conference of the North American Chapter of the Association for Computational Linguistics: Human Language Technologies (NAACL-HLT 2018).

[3] Hazarika D., Poria S., Mihalcea R., Cambria E., Zimmermann R. ICON: Interactive conversational memory network for multimodal emotion detection. Proceedings of the 2018 Conference on Empirical Methods in Natural Language Processing.

[4] Kołakowska A., Landowska A., Szwoch M., Szwoch W., Wróbel M.R. Emotion recognition and its application in software engineering. Proceedings of the 2013 6th International Conference on Human System Interactions (HSI).

[5] Eyben F., Wöllmer M., Schuller B. OpenEAR—introducing the Munich open-source emotion and affect recognition toolkit. Proceedings of the 2009 3rd International Conference on Affective Computing and Intelligent Interaction and Workshops.

[6] Poria S., Hazarika D., Majumder N., Mihalcea R. (2020). Beneath the tip of the iceberg: Current challenges and new directions in sentiment analysis research. IEEE Trans. Affect. Comput..

[7] Flynn M., Effraimidis D., Angelopoulou A., Kapetanios E., Williams D., Hemanth J., Towell T. (2020). Assessing the Effectiveness of Automated Emotion Recognition in Adults and Children for Clinical Investigation. Front. Hum. Neurosci..

[8] Wallbott H.G., Scherer K.R. (1986). Cues and channels in emotion recognition. J. Personal. Soc. Psychol..

[9] Johnson W.F., Emde R.N., Scherer K.R., Klinnert M.D. (1986). Recognition of emotion from vocal cues. Arch. Gen. Psychiatry.

[10] Sauter D.A., Panattoni C., Happé F. (2013). Children’s recognition of emotions from vocal cues. Br. J. Dev. Psychol..

[11] Adolphs R. (2002). Recognizing emotion from facial expressions: Psychological and neurological mechanisms. Behav. Cogn. Neurosci. Rev..

[12] Sprengelmeyer R., Rausch M., Eysel U.T., Przuntek H. (1998). Neural structures associated with recognition of facial expressions of basic emotions. Proc. R. Soc. Lond. Ser. B Biol. Sci..

[13] Noroozi F., Kaminska D., Corneanu C., Sapinski T., Escalera S., Anbarjafari G. (2018). Survey on emotional body gesture recognition. IEEE Trans. Affect. Comput..

[14] Saha S., Datta S., Konar A., Janarthanan R. A study on emotion recognition from body gestures using Kinect sensor. Proceedings of the 2014 International Conference on Communication and Signal Processing.

[15] Cook N.D. (2002). Tone of Voice and Mind: The Connections between Intonation, Emotion, Cognition, and Consciousness.

[16] Cook M. (1977). Gaze and Mutual Gaze in Social Encounters: How long—And when—We look others“ in the eye” is one of the main signals in nonverbal communication. Am. Sci..

[17] Merten J. (1997). Facial-affective behavior, mutual gaze, and emotional experience in dyadic interactions. J. Nonverbal Behav..

[18] Galbusera L., Finn M.T., Tschacher W., Kyselo M. (2019). Interpersonal synchrony feels good but impedes self-regulation of affect. Sci. Rep..

[19] Amiriparian S., Han J., Schmitt M., Baird A., Mallol-Ragolta A., Milling M., Gerczuk M., Schuller B. (2019). Synchronization in interpersonal speech. Front. Robot. AI.

[20] Murata A., Nomura K., Watanabe J., Kumano S. (2021). Interpersonal physiological synchrony is associated with first person and third person subjective assessments of excitement during cooperative joint tasks. Sci. Rep..

[21] Williams G.P., Kleinke C.L. (1993). Effects of mutual gaze and touch on attraction, mood, and cardiovascular reactivity. J. Res. Personal..

[22] Prochazkova E., Kret M.E. (2017). Connecting minds and sharing emotions through mimicry: A neurocognitive model of emotional contagion. Neurosci. Biobehav. Rev..

[23] Park C.Y., Cha N., Kang S., Kim A., Khandoker A.H., Hadjileontiadis L., Oh A., Jeong Y., Lee U. (2020). K-EmoCon, a multimodal sensor dataset for continuous emotion recognition in naturalistic conversations. Sci. Data.

[24] Nakatsu R., Nicholson J., Tosa N. Emotion recognition and its application to computer agents with spontaneous interactive capabilities. Proceedings of the Seventh ACM International Conference on Multimedia (Part 1).

[25] Rupauliha K., Goyal A., Saini A., Shukla A., Swaminathan S. Multimodal Emotion Recognition in Polish (Student Consortium). Proceedings of the 2020 IEEE Sixth International Conference on Multimedia Big Data (BigMM).

[26] Meng H., Romera-Paredes B., Bianchi-Berthouze N. Emotion recognition by two view SVM_2K classifier on dynamic facial expression features. Proceedings of the Face and Gesture 2011.

[27] Mehta D., Siddiqui M.F.H., Javaid A.Y. (2018). Facial emotion recognition: A survey and real-world user experiences in mixed reality. Sensors.

[28] Ozdemir M.A., Elagoz B., Alaybeyoglu A., Sadighzadeh R., Akan A. Real time emotion recognition from facial expressions using cnn architecture. Proceedings of the 2019 Medical Technologies Congress (TIPTEKNO).

[29] Liliana D.Y. (2019). Emotion recognition from facial expression using deep convolutional neural network. J. Phys. Conf. Ser..

[30] Hu M., Wang H., Wang X., Yang J., Wang R. (2019). Video facial emotion recognition based on local enhanced motion history image and CNN-CTSLSTM networks. J. Vis. Commun. Image Represent..

[31] Mehendale N. (2020). Facial emotion recognition using convolutional neural networks (FERC). SN Appl. Sci..

[32] Turan C., Lam K.M. (2018). Histogram-based local descriptors for facial expression recognition (FER): A comprehensive study. J. Vis. Commun. Image Represent..

[33] Dang K., Sharma S. Review and comparison of face detection algorithms. Proceedings of the 2017 7th International Conference on Cloud Computing, Data Science & Engineering-Confluence.

[34] Kumar A., Kaur A., Kumar M. (2019). Face detection techniques: A review. Artif. Intell. Rev..

[35] Al-Allaf O.N. (2014). Review of face detection systems based artificial neural networks algorithms. arXiv.

[36] Stąpor K. Evaluating and comparing classifiers: Review, some recommendations and limitations. Proceedings of the International Conference on Computer Recognition Systems.

[37] Mohandes M., Deriche M., Aliyu S.O. (2018). Classifiers combination techniques: A comprehensive review. IEEE Access.

[38] Jung H., Lee S., Park S., Lee I., Ahn C., Kim J. (2015). Deep temporal appearance-geometry network for facial expression recognition. arXiv.

[39] Ding H., Zhou S.K., Chellappa R. Facenet2expnet: Regularizing a deep face recognition net for expression recognition. Proceedings of the 2017 12th IEEE International Conference on Automatic Face & Gesture Recognition (FG 2017).

[40] Li S., Cui L., Zhu C., Li B., Zhao N., Zhu T. (2016). Emotion recognition using Kinect motion capture data of human gaits. PeerJ.

[41] Senecal S., Cuel L., Aristidou A., Magnenat-Thalmann N. (2016). Continuous body emotion recognition system during theater performances. Comput. Animat. Virtual Worlds.

[42] Glowinski D., Camurri A., Volpe G., Dael N., Scherer K. Technique for automatic emotion recognition by body gesture analysis. Proceedings of the 2008 IEEE Computer Society Conference on Computer Vision and Pattern Recognition Workshops.

[43] Piana S., Stagliano A., Odone F., Verri A., Camurri A. (2014). Real-time automatic emotion recognition from body gestures. arXiv.

[44] Liu Z., Zhu J., Bu J., Chen C. (2015). A survey of human pose estimation: The body parts parsing based methods. J. Vis. Commun. Image Represent..

[45] Zhang H.B., Lei Q., Zhong B.N., Du J.X., Peng J. (2016). A survey on human pose estimation. Intell. Autom. Soft Comput..

[46] Zheng C., Wu W., Yang T., Zhu S., Chen C., Liu R., Shen J., Kehtarnavaz N., Shah M. (2020). Deep Learning-Based Human Pose Estimation: A Survey. arXiv.

[47] Aracena C., Basterrech S., Snáel V., Velásquez J. Neural networks for emotion recognition based on eye tracking data. Proceedings of the 2015 IEEE International Conference on Systems, Man, and Cybernetics.

[48] Wu S., Du Z., Li W., Huang D., Wang Y. Continuous emotion recognition in videos by fusing facial expression, head pose and eye gaze. Proceedings of the 2019 International Conference on Multimodal Interaction.

[49] Swain M., Routray A., Kabisatpathy P. (2018). Databases, features and classifiers for speech emotion recognition: A review. Int. J. Speech Technol..

[50] Khalil R.A., Jones E., Babar M.I., Jan T., Zafar M.H., Alhussain T. (2019). Speech emotion recognition using deep learning techniques: A review. IEEE Access.

[51] Chandrasekar P., Chapaneri S., Jayaswal D. Automatic speech emotion recognition: A survey. Proceedings of the 2014 International Conference on Circuits, Systems, Communication and Information Technology Applications (CSCITA).

[52] El Ayadi M., Kamel M.S., Karray F. (2011). Survey on speech emotion recognition: Features, classification schemes, and databases. Pattern Recognit..

[53] Jahangir R., Teh Y.W., Hanif F., Mujtaba G. (2021). Deep learning approaches for speech emotion recognition: State of the art and research challenges. Multimed. Tools Appl..

[54] Lin Y.S., Lee C.C. Deriving Dyad-Level Interaction Representation Using Interlocutors Structural and Expressive Multimodal Behavior Features. Proceedings of the INTERSPEECH.

[55] Lee C.C., Busso C., Lee S., Narayanan S.S. Modeling mutual influence of interlocutor emotion states in dyadic spoken interactions. Proceedings of the Tenth Annual Conference of the International Speech Communication Association.

[56] Yeh S.L., Lin Y.S., Lee C.C. An interaction-aware attention network for speech emotion recognition in spoken dialogs. Proceedings of the ICASSP 2019–2019 IEEE International Conference on Acoustics, Speech and Signal Processing (ICASSP).

[57] Castellano G., Kessous L., Caridakis G. (2008). Emotion recognition through multiple modalities: Face, body gesture, speech. Affect and Emotion in Human-Computer Interaction.

[58] Wang X., Chen X., Cao C. (2020). Human emotion recognition by optimally fusing facial expression and speech feature. Signal Process. Image Commun..

[59] Majumder N., Hazarika D., Gelbukh A., Cambria E., Poria S. (2018). Multimodal sentiment analysis using hierarchical fusion with context modeling. Knowl. Based Syst..

[60] Pan Z., Luo Z., Yang J., Li H. (2020). Multi-modal attention for speech emotion recognition. arXiv.

[61] Busso C., Bulut M., Lee C.C., Kazemzadeh A., Mower E., Kim S., Chang J.N., Lee S., Narayanan S.S. (2008). IEMOCAP: Interactive emotional dyadic motion capture database. Lang. Resour. Eval..

[62] Posner J., Russell J.A., Peterson B.S. (2005). The circumplex model of affect: An integrative approach to affective neuroscience, cognitive development, and psychopathology. Dev. Psychopathol..

[63] Wiem M.B.H., Lachiri Z. (2017). Emotion classification in arousal valence model using MAHNOB-HCI database. Int. J. Adv. Comput. Sci. Appl..

[64] LeCun Y., Haffner P., Bottou L., Bengio Y. (1999). Object recognition with gradient-based learning. Shape, Contour and Grouping in Computer Vision.

[65] Zeiler M.D., Krishnan D., Taylor G.W., Fergus R. Deconvolutional networks. Proceedings of the 2010 IEEE Computer Society Conference on Computer Vision and Pattern Recognition.

[66] He K., Zhang X., Ren S., Sun J. Deep residual learning for image recognition. Proceedings of the IEEE Conference on Computer Vision and Pattern Recognition.

[67] Lea C., Flynn M.D., Vidal R., Reiter A., Hager G.D. Temporal convolutional networks for action segmentation and detection. Proceedings of the IEEE Conference on Computer Vision and Pattern Recognition.

[68] Shen C. (2015). Analysis of detrended time-lagged cross-correlation between two nonstationary time series. Phys. Lett. A.

[69] Vaswani A., Shazeer N., Parmar N., Uszkoreit J., Jones L., Gomez A.N., Kaiser L., Polosukhin I. (2017). Attention is all you need. arXiv.

[70] Ishida T., Yamane I., Sakai T., Niu G., Sugiyama M. (2020). Do We Need Zero Training Loss After Achieving Zero Training Error?. arXiv.

[71] Farooq M., Hussain F., Baloch N.K., Raja F.R., Yu H., Zikria Y.B. (2020). Impact of Feature Selection Algorithm on Speech Emotion Recognition Using Deep Convolutional Neural Network. Sensors.

[72] Haq S., Jackson P.J., Edge J. Speaker-dependent audio-visual emotion recognition. Proceedings of the AVSP.

[73] Ando S., Huang C.Y. Deep over-sampling framework for classifying imbalanced data. Proceedings of the Joint European Conference on Machine Learning and Knowledge Discovery in Databases.

[74] Buda M., Maki A., Mazurowski M.A. (2018). A systematic study of the class imbalance problem in convolutional neural networks. Neural Netw..

[75] Pouyanfar S., Tao Y., Mohan A., Tian H., Kaseb A.S., Gauen K., Dailey R., Aghajanzadeh S., Lu Y.H., Chen S.C. Dynamic sampling in convolutional neural networks for imbalanced data classification. Proceedings of the 2018 IEEE Conference on Multimedia Information Processing and Retrieval (MIPR).

[76] Cao K., Wei C., Gaidon A., Arechiga N., Ma T. (2019). Learning imbalanced datasets with label-distribution-aware margin loss. arXiv.

[77] Dong Q., Gong S., Zhu X. (2018). Imbalanced deep learning by minority class incremental rectification. IEEE Trans. Pattern Anal. Mach. Intell..

[78] Khan S., Hayat M., Zamir S.W., Shen J., Shao L. Striking the right balance with uncertainty. Proceedings of the IEEE/CVF Conference on Computer Vision and Pattern Recognition.

[79] Lee P.H. (2014). Resampling methods improve the predictive power of modeling in class-imbalanced datasets. Int. J. Environ. Res. Public Health.

[80] Zeng H., Li X., Borghini G., Zhao Y., Aricò P., Di Flumeri G., Sciaraffa N., Zakaria W., Kong W., Babiloni F. (2021). An EEG-Based Transfer Learning Method for Cross-Subject Fatigue Mental State Prediction. Sensors.

[81] Arrieta A.B., Díaz-Rodríguez N., Del Ser J., Bennetot A., Tabik S., Barbado A., García S., Gil-López S., Molina D., Benjamins R. (2020). Explainable Artificial Intelligence (XAI): Concepts, taxonomies, opportunities and challenges toward responsible AI. Inf. Fusion.

